# Critical Role for G_i/o_-Protein Activity in the Dorsal Striatum in the Reduction of Voluntary Alcohol Intake in C57Bl/6 Mice

**DOI:** 10.3389/fpsyt.2018.00112

**Published:** 2018-04-05

**Authors:** Meridith T. Robins, Terrance Chiang, Kendall L. Mores, Doungkamol Alongkronrusmee, Richard M. van Rijn

**Affiliations:** ^1^Department of Medicinal Chemistry and Molecular Pharmacology, Purdue University, West Lafayette, IN, United States; ^2^Purdue University Institute for Integrative Neuroscience, Purdue University, West Lafayette, IN, United States

**Keywords:** dorsal striatum, alcohol intake, biased signaling, delta-opioid receptor, β-arrestin, designer receptors exclusively activated by designer drugs, C57BL/6 mice

## Abstract

The transition from non-dependent alcohol use to alcohol dependence involves increased activity of the dorsal striatum. Interestingly, the dorsal striatum expresses a large number of inhibitory G-protein-coupled receptors (GPCRs), which when activated may inhibit alcohol-induced increased activity and can decrease alcohol consumption. Here, we explore the hypothesis that dorsal striatal G_i/o_-protein activation is sufficient to reduce voluntary alcohol intake. Using a voluntary, limited-access, two-bottle choice, drink-in-the-dark model of alcohol (10%) consumption, we validated the importance of G_i/o_ signaling in this region by locally expressing neuron-specific, adeno-associated-virus encoded G_i/o_-coupled muscarinic M_4_ designer receptors exclusively activated by designer drugs (DREADD) in the dorsal striatum and observed a decrease in alcohol intake upon DREADD activation. We validated our findings by activating G_i/o_-coupled delta-opioid receptors (DORs), which are natively expressed in the dorsal striatum, using either a G-protein biased agonist or a β-arrestin-biased agonist. Local infusion of TAN-67, an *in vitro*-determined G_i/o_-protein biased DOR agonist, decreased voluntary alcohol intake in wild-type and β-arrestin-2 knockout (KO) mice. SNC80, a β-arrestin-2 biased DOR agonist, increased alcohol intake in wild-type mice; however, SNC80 decreased alcohol intake in β-arrestin-2 KO mice, thus resulting in a behavioral outcome generally observed for G_i/o_-biased agonists and suggesting that β-arrestin recruitment is required for SNC80-increased alcohol intake. Overall, these results suggest that activation G_i/o_-coupled GPCRs expressed in the dorsal striatum, such as the DOR, by G-protein biased agonists may be a potential strategy to decrease voluntary alcohol consumption and β-arrestin recruitment is to be avoided.

## Introduction

Alcoholism and alcohol abuse is a widespread health issue, placing a large burden at both the individual and societal level. Yet, pharmacological treatment options are still limited. Currently, only three drugs have been approved by the Food and Drug Administration for the treatment of alcohol use disorders (AUD), and each come with their own limitations in therapeutic efficacy (1); therefore, it is imperative to identify novel targets for more effective drug development, with hopes of increasing the number of treatment options and compliance for AUD management.

One potential AUD treatment approach is to increase inhibition of the dorsal striatum, a brain region with observed increasing activation upon alcohol tasting in heavy alcohol drinking human subjects (2). In contrast to the ventral striatum, which is implicated in reward-associated learning and behavior, the dorsal striatum is heavily involved in the transition to compulsive drug or alcohol seeking and taking (2–5). In rats, habitual alcohol self-administration increases habit-like responding with decreased sensitivity to alcohol devaluation (6). This shift toward habit-like responding, as well as reports of increased hyperexcitability and altered glutamatergic and GABAergic transmission in the dorsomedial striatum upon alcohol exposure (7–9), suggests molecular alterations in this brain region lead to behavioral reinforcement of alcohol intake resulting in habitual, excessive alcohol intake (3, 7, 9). We hypothesized that one conceivable strategy to inhibit this alcohol-induced neuronal excitability is by activation of metabotropic, inhibitory G_i/o_-protein signaling pathways *via* G-protein-coupled receptors (GPCRs) expressed on neurons in this region.

Interestingly, a large number of GPCRs expressed in the dorsal striatum couple to inhibitory G proteins (G_i/o_) (10, 11), thereby providing an ideal target for inhibiting this hyperexcitability observed in the dorsal striatum following persistent alcohol use. To investigate our hypothesis, G_i/o_-coupled designer receptors exclusively activated by designer drugs (DREADDs) can provide powerful tools (12, 13) to increase G_i/o_ signaling in a specific brain region, such as the dorsal striatum, on an experimenter’s predetermined time point to determine the role of the dorsal striatum in modulating alcohol consumption. In addition to artificially increasing G_i/o_ signaling using viral DREADD strategies, agonists have been developed to preferentially activate G_i/o_-protein signal pathways over the competing β-arrestin recruitment and signaling pathways for receptors endogenously expressed in the dorsal striatum, with recent advances in opioid receptor pharmacology being a prime example (14–16). For this study, the delta-opioid receptor (DOR), a G_i/o_-coupled GPCR with strong expression in the dorsal striatum (17), provided us with a powerful tool to investigate our hypothesis that G_i/o_ signaling in the dorsal striatum can reduce alcohol use. DORs are thought to play a protective role in AUD, as DOR knockout (KO) mice display increased alcohol consumption and preference compared with wild-type, kappa-, or mu-opioid receptor KO mice, suggesting that DOR expression prevents escalated alcohol intake compared with other opioid receptor subtypes (18). Moreover, DORs are heavily expressed in the dorsal striatum presynaptically on corticostriatal glutamatergic inputs (19), both pre- and postsynaptically on interneurons within this brain region, and enriched on D_2_ receptor-expressing MSNs (as compared with D_1_ receptor-expressing MSNs) (20–22). Furthermore, direct activation (23) or indirect activation of DORs *via* alcohol-induced release of endogenous enkephalins (24) in the dorsal striatum induces long-term depression (LTD).

The importance of the activation of dorsal striatal DORs in the modulation of alcohol intake was first evident in a report by Nielsen et al., where infusion of the DOR-selective agonist SNC80 into the dorsal striatum increased alcohol intake in rats while the DOR antagonist naltrindole reduced intake (25). This finding that DOR agonist SNC80 increased alcohol was somewhat surprising as DOR expression was previously mentioned to be protective against increased alcohol intake (18). Yet, our recent work investigating a panel of DOR agonists suggests that SNC80 prefers to recruit β-arrestin protein through a mechanism called biased signaling (also termed functional selectivity) (26, 27), where we have additionally correlated *in vitro* β-arrestin recruitment with *in vivo* increased alcohol intake (28). In that same study investigating the behavioral effects of DOR biased signaling, we also observed that DOR agonists that weakly recruit β-arrestin, particularly TAN-67 (and thus are G-protein-biased), decreased alcohol intake in mice in a limited-access, drinking-in-the-dark (DID) protocol to 10% alcohol (28).

Therefore, here we hypothesized that activation of G_i/o_ signaling in the dorsal striatum would be beneficial in reducing alcohol intake, whereas β-arrestin signaling will lead to enhanced alcohol use. To begin to investigate this hypothesis, we first utilized hM_4_Di DREADD technology (12) to identify the broad role of G_i/o_-coupled receptor activation in the dorsal striatal on voluntary alcohol intake in C57Bl/6 male mice using a two-bottle choice, limited-access DID protocol. In addition, we selectively infused our previously identified differentially biased DOR agonists in wild-type and β-arrestin-2 KO mice to more specifically investigate the effect of increased dorsal striatal DOR G_i/o_-protein signaling (versus β-arrestin) on voluntary alcohol intake.

## Materials and Methods

### Drugs and Chemicals

SNC80 and SB205607 (TAN-67) were purchased from Tocris, R&D systems (Minneapolis, MN, USA); naltrindole hydrochloride, forskolin, 200 proof ethyl alcohol, leu-enkephalin, sodium chloride, DMSO, cocaine hydrochloride, and clozapine-*N*-oxide (CNO) were purchased from Sigma-Aldrich (St. Louis, MO, USA). For dorsal striatal infusion studies, TAN-67 and SNC80 were diluted in 0.9% saline to a concentration of 10 µM; for cellular assays, drugs were dissolved in water. Cocaine was dissolved in 0.9% saline for an administered dose of 15 mg/kg, and CNO was dissolved in 100% DMSO and diluted to a concentration of 0.2 mg/ml in saline (final DMSO concentration of 0.5% and administered dose of 2 mg/kg). Both cocaine and CNO were injected intraperitoneally (i.p.) during experimentation. Non-Cre-dependent AAV8-hSyn-hM_4_Di-mCherry (7.4 × 10^12^ vg/ml) virus and AAV8-hSyn-EGFP (3.9 × 10^12^ vg/ml) virus were obtained from the University of North Carolina Vector Core. Both viruses were chosen as they specifically express in neurons through use of the synapsin promoter. A 100 mg/kg ketamine (Henry Schein, Dublin, OH, USA):10 mg/kg xylazine (Sigma-Aldrich) cocktail was administered to induce anesthesia for cannulation surgeries and before transcardial perfusion. All systemic drugs were injected at a volume of 10 ml/kg.

### Cell Culture and Biased Signaling Assays

Competition binding assays were performed using the Tag-lite assay according to the manufacturer’s protocol (Cis-Bio, Bedford, MA, USA). In short, Tb-labeled HEK293-SNAP-hDOR cells/well (4,000 cells/well) were plated in 10 µl Tag-lite medium into low-volume 384-well plates in the presence of 5 µl 8 nM fluorescent naltrexone (final concentration) and 5 µl of an increasing concentration of TAN-67, leu-enkephalin, or SNC80 and incubated at room temperature for 3 h. cAMP inhibition and β-arrestin-2 recruitment assays were performed as previously described (28). In brief, for cAMP inhibition assays HEK293 (Life Technologies, Grand Island, NY, USA) cells (15,000 cells/well, 7.5 µl) transiently expressing FLAG-mDOR (29, 30), SNAP-rDOR, or SNAP-hDOR (Cis-Bio), and pGloSensor22F-cAMP plasmids (Promega, Madison, WI, USA) were incubated with Glosensor reagent (Promega, 7.5 µl, 2% final concentration) for 90 min at 37°C/5% CO_2_. Cells were stimulated with 5 µl DOR agonist 20 min before 30 µM forskolin (5 µl) stimulation for an additional 15 min. For β-arrestin-2 recruitment assays, CHO-hDOR PathHunter β-arrestin-2 cells (DiscoverX, Fremont, CA, USA) were plated (2,500 cells/well, 10 µl) before stimulation with 2.5 µl DOR agonists for 90 min at 37°C/5% CO_2_, after which cells were incubated with 6 µl cell assay buffer for 60 min at room temperature as per the manufacturer’s protocol. Luminescence and fluorescence for each of the assays were measured using a FlexStation3 plate reader (Molecular Devices, Sunnyvale, CA, USA).

### SNAP-rDOR Construction

Rat DOR cDNA was amplified from the pUC17-rDOR plasmid (Versaclone cDNA NP_036749, R&D systems) using the following forward (5′-CTTCGATATCTTGGAGCCGGTGCCTTCTG-3′) and a standard M13 reverse primer using the Pfu Ultra II Hotstart PCR Mastermix (Agilent, Santa Clara, CA, USA) according to the manufacturer’s protocol. The amplified rDOR PCR product and the pSNAP-hDOR plasmid (Cis-Bio) were restricted using EcoRV and XhoI restriction enzymes (New England BioLabs, Ipswich, MA, USA), and the rDOR construct was exchanged with the hDOR gene followed by ligation with T4 DNA Ligase (New England BioLabs) and transformation into NEB5α competent cells (New England BioLabs). The SNAP-rDOR was fully sequenced to ensure correct orientation and absence of point mutations introduced during amplification.

### Animals

37 male C57BL/6 mice (age 6 weeks) were purchased from Harlan and habituated for to the facility 1 week before surgery. For β-arrestin-2 KO animals, animals were bred in house, and 16 animals were selected for surgery [for complete details on strain origin see Ref. (28)]. Throughout the experiment, animals were kept in at ambient temperature of 21°C in a room maintained on a reversed 12L:12D cycle (lights off at 10:00, lights on at 22:00) in Purdue University’s animal facility, which is accredited by the Association for Assessment and Accreditation of Laboratory Animal Care. This study was carried out in accordance with the recommendations of the National Institutes of Health Guide for the Care and Use of Laboratory Animals. The protocol (#1305000864) was approved by the Purdue University Institutional Animal Care and Use Committee.

### Surgical Cannulation

Directly before surgery, mice were anesthetized with ketamine/xylazine (i.p.). A Kopf model 1900 stereotaxic alignment system (David Kopf Instruments, Tujunga, CA, USA) was used to drill two holes using Kyocera #69 drill bits at the following coordinates from bregma: AP = +1 mm, ML = ±1.5 mm, DV: −3.25 mm (31, 32). For experiments involving drug infusion, an additional two holes were drilled using Kyocera #60 drill bits at the following coordinates from bregma: AP = −2.4 mm, ML = ±1.6 mm, and 1 mm screws were positioned to ensure head-cap stability. A bilateral 22-gage guide cannula (cut 1.5 mm below pedestal, PlasticsOne, Roanoke, VA, USA) was attached to the skull using Geristore dental cement (DenMat, Lompoc, CA, USA). In total, two animals did not wake up from surgery, and eight animals were euthanized after their cannulas came off postoperation or throughout alcohol training and/or experimentation.

### Viral Injection

After cannulation surgery, animals were single housed in double grommet cages to allow recovery and individual measurement of fluid intake. One-week postsurgery, mice were anesthetized as previously described and injected bilaterally with 450 nl of virus using a Harvard Apparatus infusion pump at a speed of 50 nl/min *via* internal cannula with 0.5 mm projection (PlasticsOne). The internal cannula was left in place for an additional 5 min to allow viral dispersion and prevent backflow of the viral solution into the injection syringe. All biohazard work was approved by the Institutional Biohazard Committee at Purdue University (#13-013-16).

### Voluntary Alcohol Intake

One-week postsurgery and/or 1-week post-viral injection, mice were exposed to a limited-access (4 h/day), two-bottle choice (water versus 10% ethanol), DID protocol 3 h into their active cycle (dark phase) until the alcohol intake was stable as previously described (29). This model has previously shown that TAN-67 administration before the 4-h session decreases alcohol intake with a correlated decrease in blood ethanol concentration (with no TAN-67 effects on alcohol metabolism) (29). Mice were trained for 3 weeks during which the mice initially increased their alcohol intake before reaching steady state consumption. Bottle weights were measured directly before and after the 4-h access period to the second decimal point to determine fluid intake during this access period. Weights of bottles were corrected for any spillage with fluid bottles placed on empty cages.

### Drug Infusion or Injection

After 3 weeks of exposure to the drinking model described earlier, alcohol and water intake on the day of infusion (Friday) was compared with the average alcohol intake over the preceding 3 days (Tuesday–Thursday) to determine if either drug injection or infusion altered voluntary alcohol intake in the following manipulations. For experiments involving viral expression, the AAV injected mice were injected with i.p. saline (with 0.5% DMSO) for vehicle measurements in week 4 and 2 mg/kg CNO (i.p.) the following week (week 5). The dose of CNO of 2 mg/kg was utilized as it has previously been shown to be effective in mice in activating expressed DREADDs (33, 34). Also, this relatively low dose limits high concentrations of clozapine caused by metabolism of CNO (35). For experiments involving direct drug infusion into the dorsal striatum, animals received a 150 nl bilateral infusion of saline into the dorsal striatum on Friday of the fourth week of alcohol exposure. In weeks 5 and 6, animals received either a 150 nl infusion of 10 µM TAN-67 or SNC80, respectively, thereby allowing for a within subjects’ analysis. The order of the drug infusions was chosen to mitigate potential DOR internalization and/or degradation as SNC80 is a high internalizing agonist *in vitro* and *in vivo* (36, 37). Doses of TAN-67 and SNC80 were determined based on previous studies of SNC80 infusions in rats (25) and *in vivo* specificity of TAN-67 and SNC80 for the DOR over MOR or KOR had been previously established using KO animals (29, 38). Importantly, no seizure behavior was observed up SNC80 infusion (39) following any dorsal striatal infusions.

### Locomotor Activity

Square locomotor boxes from Med Associates (*L* 27.3 cm × *W* 27.3 cm × *H* 20.3 cm, St. Albans, VT, USA) were used to monitor locomotor activity during the active/dark phase as previously described (28). For AAV experiments, animals were placed in the locomotor box 15 min before CNO (2 mg/kg, i.p.) injection to allow baseline locomotor activity scoring. After 15 min, all animals were injected with CNO and then placed back into the box for an additional 60 min of testing to measure the total distance traveled in 60 min following drug injection. For intra-dorsal striatal infusion of SNC80, animals received either 10 µM SNC80 or vehicle (saline 0.9%) infusion and were placed immediately in the boxes for 90 min; locomotor data were analyzed 30 min after drug infusion as this is when drinking experiments began in the previously described alcohol intake studies.

### Cannula Location and Immunohistochemical Analysis

For animals undergoing drug infusions, animals were sacrificed *via* transcardial perfusion within 1 week following their final drinking session. During analysis, it was determined that the cannula of one mouse from these experiments was not positioned properly and this animal was removed from analysis (placement was too ventral). Cannulation location and viral expression was verified with confocal microscopy (Nikon A1, Nikon, Melville, NY, USA) with an area of capture of 1.69 mm^2^. The experimenter performing analysis was blind to the experimental conditions; all images were evaluated in greyscale to prevent unintentional bias.

### Cocaine-Induced c-Fos Activation in DREADD-Expressing Animals

For viral expression studies, both groups of mice were injected with 2 mg/kg CNO (i.p.) during the dark/active phase for each animal. Twenty minutes later, animals were injected with 15 mg/kg cocaine (i.p.) to induce expression of immediate-early gene c-Fos. Brains were collected 90 min following cocaine exposure *via* transcardial perfusion. Extracted brain samples embedded and frozen in Tissue-Tek^®^ O.C.T. compound (VWR, Radnor, PA, USA) in tissue molds (VWR) and sliced into 50 µm coronal sections *via* cryostat (Leica Microsystems Inc., Buffalo Grove, IL, USA). Immunohistochemical staining was conducted using primary rabbit anti-c-Fos antibody (sc-52, Santa Cruz Biotechnology, Dallas, TX, USA), diluted 1:1,000. Control-GFP animal brains were applied Alexa-Fluor 594 goat anti-rabbit antibody (A-11012, Life Technologies, Grand Island, NY, USA) diluted 1:1,000. hM_4_Di-mCherry animal brains were applied Alexa-Fluor 488 goat anti-rabbit (A-11008, Life Technologies, Grand Island, NY, USA) diluted 1:1,000. Brain slices were mounted onto microscope slides (Fisher Scientific, Hampton, NH, USA) for confocal microscopy with an area of the capture of 0.40 mm^2^. Images were processed using ImageJ software (NIH, Bethesda, MD, USA) for the number of c-Fos positive cells in the dorsal striatum surrounding the viral injection site in infected cell populations. The experimenter performing analysis was blind to the experimental conditions; all images were evaluated in greyscale to prevent unintentional bias.

### Statistical Analysis

All data are presented as means ± SEM and was performed using GraphPad Prism7 software (GraphPad Software, La Jolla, CA, USA). Differences between control-GFP and hM_4_Di-mCherry animals were analyzed by student two-tailed *t*-test for differences in baseline water intake, alcohol intake, alcohol preference, locomotion after CNO injection, and c-Fos expression in the dorsal striatum. Differences in alcohol intake after saline injection and CNO injection were evaluated by repeated measures, multiple comparisons (Bonferroni) two-way ANOVA. For *in vitro* assays, non-linear regression using a dose–response to either inhibition (binding, cAMP) or stimulation (β-arrestin-2 recruitment) was conducted to determine pIC50 or pEC50, respectively. In direct dorsal striatal drug infusion experiments, differences in voluntary alcohol intake, water intake, and alcohol preference were analyzed by repeated measures, multiple comparisons (Tukey) two-way ANOVA. The Grubb’s outlier test (alpha = 0.05) was used to identify potential outliers throughout the study. Statistical analysis was conducted in guidance with and approved by Purdue University’s Department of Statistics.

## Results

### Activation of a G_i/o_-Coupled DREADD in the Dorsal Striatum Decreases Alcohol Intake

**Figure 1:**
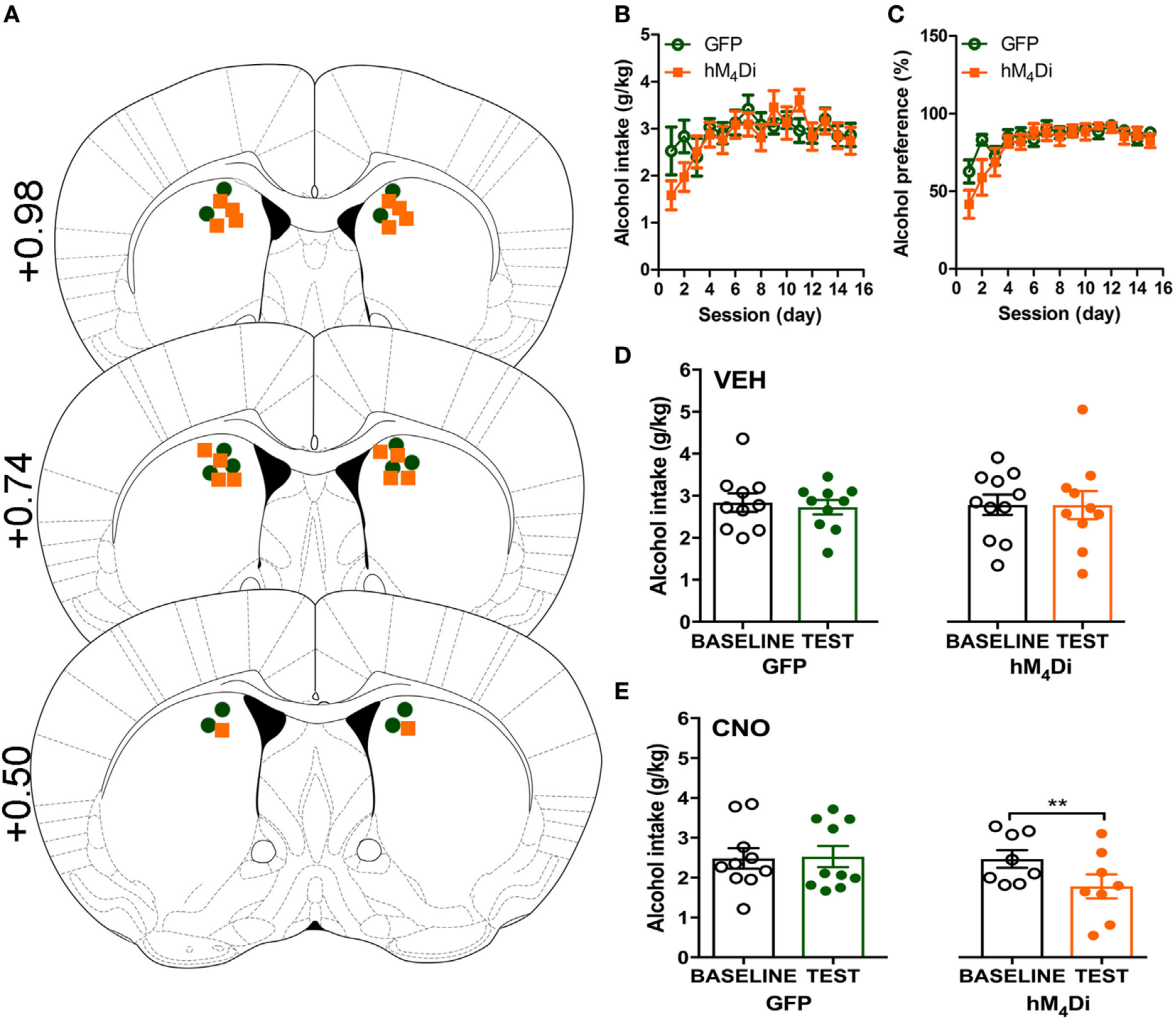
Activation of virally expressed hM_4_Di in the dorsal striatum decreases alcohol intake in mice. Cannula placement was verified for all animals included in behavioral analysis **(A)**. C57BL/6 mice (*n* = 10–11) injected in the dorsal striatum with either AAV8-hSyn-EGFP (GFP) or AAV8-hSyn-hM_4_Di-mCherry (hM_4_Di) were trained to consume alcohol in a two-bottle, drinking-in-the-dark protocol. Both groups of animals displayed a similar increase in alcohol intake **(B)** and preference **(C)**. Vehicle injection (saline 0.9%, i.p.) did not change alcohol intake **(D)**. Systemic clozapine-*N*-oxide (CNO) injection (2 mg/kg i.p.) significantly decreased alcohol intake in mice expressing hM_4_Di, but not GFP, in the dorsal striatum **(E)**. Significance by unpaired, Student’s *t*-test for AUC or two-way ANOVA with Bonferroni posttest for matching, **p < 0.01.

Cannula placement was verified postmortem (*n* = 10–11) through immunohistochemical analysis of viral expression (Figure 1A). Viral infusions of control-GFP (green fluorescent protein) or hM_4_Di-mCherry in the dorsal striatum did not alter baseline alcohol intake, water intake, or alcohol preference when comparing the two groups [Figure 1B, *t*(20) = 0.81, *p* = 0.32; Figure 1C, *t*(20) = 0.60, *p* = 0.42; Figure S1A in Supplementary Material, *t*(20) = 1.01, *p* = 0.55]. Vehicle injection (0.5% DMSO, i.p.) did not affect alcohol intake for control or hM_4_Di-expressing animals in alcohol intake (Figure 1D; see Table 1 for full statistical analysis for experimental group), water intake (Figure S1B in Supplementary Material), or alcohol preference in control-GFP or hM_4_Di-mCherry mice (Figure S1E in Supplementary Material). Unlike saline injection, CNO injection (2 mg/kg, i.p.) significantly reduced alcohol intake in hM_4_Di-expressing mice compared with GFP-control, as evaluated by two-way ANOVA (Figure 1E; Figure S1D in Supplementary Material, effect of drug × virus: *p* = 0.03), where Bonferroni posttest analysis revealed that CNO significantly reduced alcohol intake only in hM_4_Di-expressing animals and not control-GFP-expressing mice (*p* < 0.002). No significant change in water intake was observed after CNO injection in the testing period for in either group of animals (Figure S1C in Supplementary Material). CNO injection did not alter alcohol preference in control-GFP or hM_4_Di-mCherry mice (Figure S1F in Supplementary Material).

**Table 1:** Analysis of alcohol-related behaviors in control-GFP versus hM_4_Di-mCherry designer receptors exclusively activated by designer drugs (DREADD)-expressing mice.

	df	Alcohol intake	Water intake	Alcohol preference
Baseline^a^ (Student’s *t*-test)	20	*t* = 0.812 *p* = 0.32	*t* = 0.603 *p* = 0.42	*t* = 1.01 *p* = 0.55
**Vehicle**^^b^^				
Drug	1, 19	*F* = 0.18 *p* = 0.86	*F* = 0.52 *p* = 0.48	*F* = 3.82 *p* = 0.07
Virus		*F* = 0.04 *p* = 0.85	*F* = 0.00 *p* = 0.96	*F* = 0.26 *p* = 0.42
Drug × virus		*F* = 0.00 *p* = 0.99	*F* = 0.26 *p* = 0.61	*F* = 2.85 *p* = 0.11
**Clozapine-*N*-oxide**^^b^^				
Drug	1, 19	*F* = 4.00 *p* = 0.06	*F* = 1.20 *p* = 0.29	*F* = 0.42 *p* = 0.52
Virus		*F* = 1.26 *p* = 0.28	*F* = 2.37 *p* = 0.14	*F* = 0.90 *p* = 0.35
Drug × virus		*F* = 5.17 *p* = 0.03 Control versus DREADD *p* < 0.002	*F* = 0.28 *p* = 0.60	*F* = 1.01 *p* = 0.33

*^a^Student’s t-test*.

*^b^Two-way, repeated measures (Bonferroni) ANOVA*.

**Figure 2:**
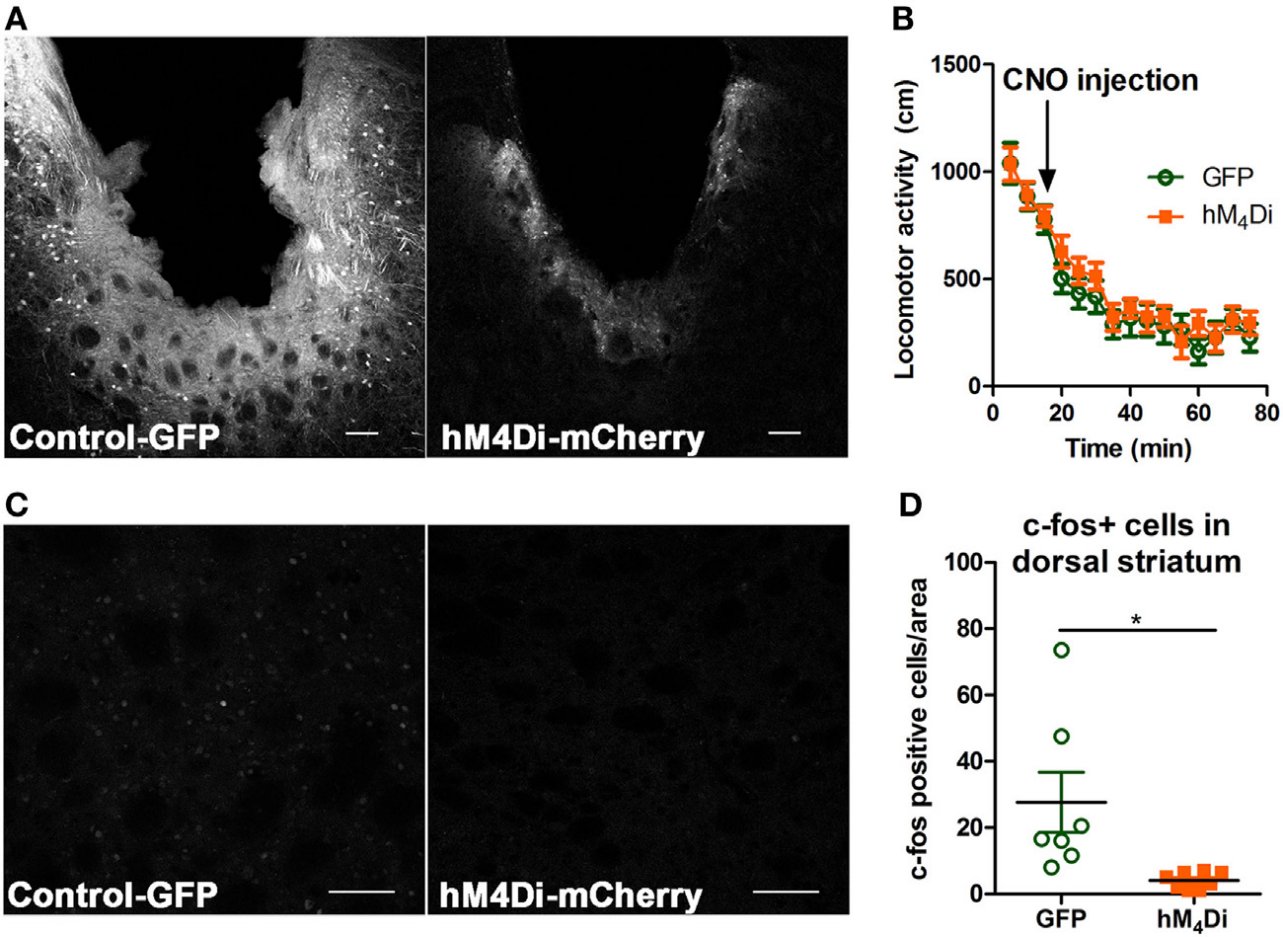
Verification of viral expression and functionality of control-GFP or hM_4_Di-mCherry in the dorsal striatum. Viral expression verification *via* confocal microscopy of control-GFP (left) and hM_4_Di-mCherry (right) in the dorsal striatum (scale bar = 100 µm) **(A)**. C57BL/6 mice (*n* = 10–11 per group) expressing GFP or hM_4_Di in the dorsal striatum did not display significant clozapine-*N*-oxide (CNO) (2 mg/kg, i.p.) induced locomotor activity in the 60-min session after CNO injection **(B)**. Immunohistochemical representation of c-Fos activation in the dorsal striatum in animals expressing control-GFP (left) and hM_4_Di-mCherry (right) in the dorsal striatum (scale bar = 100 µm) **(C)**. Decreased c-Fos expression in dorsal striatum after cocaine challenge (15 mg/kg, i.p.) in C57BL/6 mice (*n* = 7–8) expressing hM_4_Di-mCherry versus control-GFP observed confocal microscopy **(D)**. Significance by unpaired two-tailed *t*-test, *p < 0.05.

Both viruses properly expressed in the dorsal striatum (Figure 2A). Differences in visualization of the control-GFP and hM_4_Di-mCherry expression may potentially result from differences in viral load and protein expression or inherent differences in quantum yield and extinction coefficients between GFP and mCherry (40). Considering that the striatum is part of the basal ganglia that controls movement (6, 41), we determined whether CNO activation of dorsal striatal hM_4_Di altered locomotor activity where we observed that CNO did not alter locomotor activity between control-GFP and hM_4_Di-mCherry expressing mice in a 60-min locomotor period after injection [Figure 2B, *t*(19) = 0.78, *p* = 0.45]. To confirm the inhibitory functionality of hM_4_Di expression, we determined if CNO activation of hM_4_Di could prevent cocaine-induced c-Fos expression (42, 43), an acceptable approach previously used in other studies to validate functionality of inhibitory DREADDs (42–45). We observed that activation of striatal hM_4_Di with CNO (2 mg/kg, i.p.) before a cocaine challenge (15 mg/kg, i.p.) significantly inhibited c-Fos activation in animals expressing hM_4_Di versus GFP controls (control were also administered CNO before cocaine challenge) [Figures 2C,D; *t*(13) = 2.78, *p* < 0.02], suggesting that activation of hM_4_Di *via* CNO before cocaine challenge inhibited cAMP pathway activity by G_i/o_-coupled inhibition. The variability in c-Fos expression in control-GFP may be a result of intrinsic differences in response to psychostimulants between animals, which has been commonly observed in C57Bl/6 mice (46).

### *In Vitro* Characterization of the β-Arrestin-2 Biased DOR Agonist, SNC80

**Figure 3:**
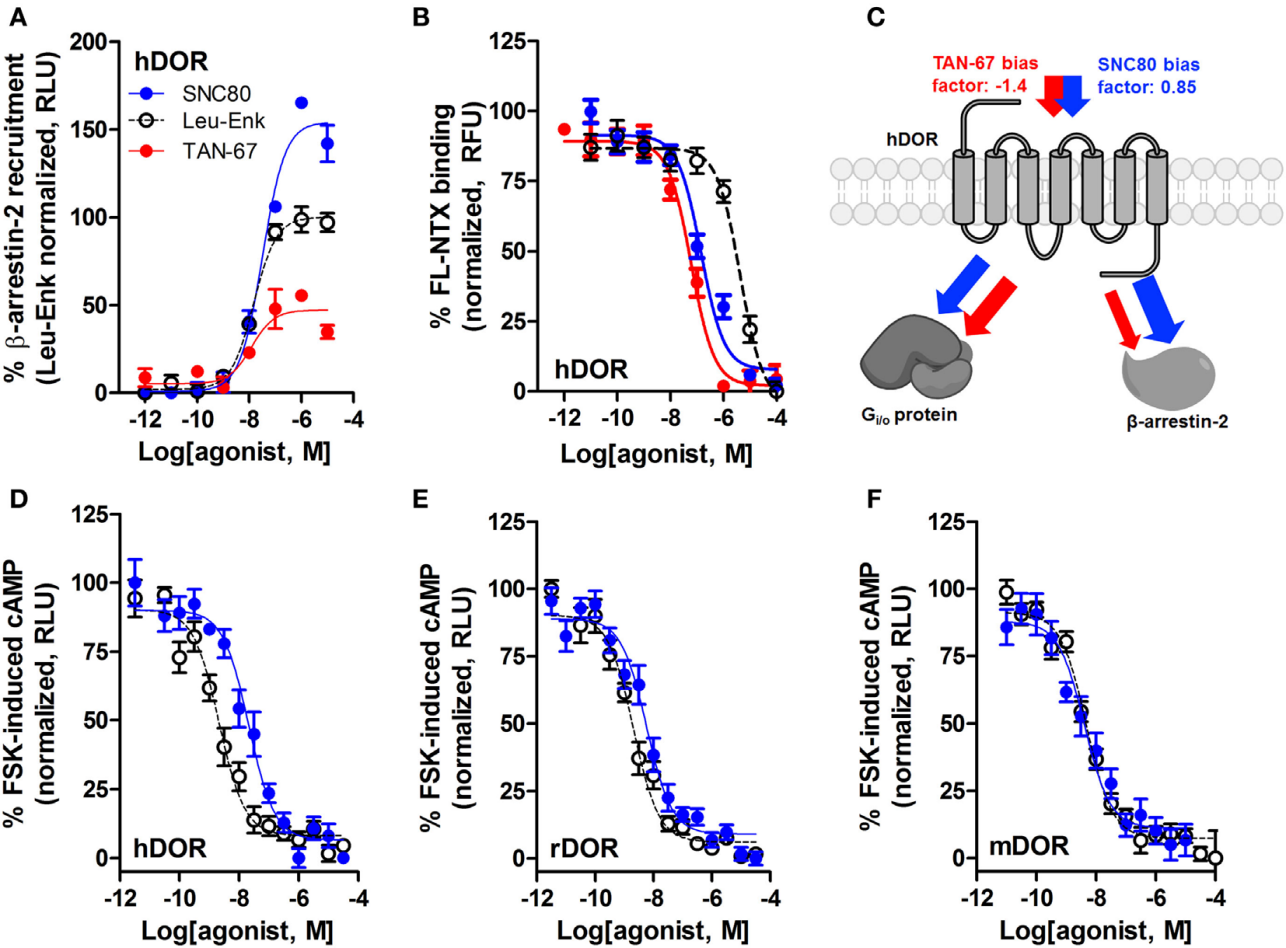
SNC80 is a β-arrestin-biased agonist with comparable potency across species in heterologous cell systems. At the hDOR, SNC80 acts as a β-arrestin-2 super-agonist compared with the endogenous agonist leu-enkephalin and the weak β-arrestin-2 recruiter TAN-67 **(A)**. SNC80 and TAN-67 bind to hDOR with similar affinity **(B)**. Schematic representation of the observed ligand bias of TAN-67 and SNC80 at hDOR, with calculated bias factor **(C)**. SNC80 and Leu-enkephalin have similar potency to inhibit forskolin-induced cAMP production at hDOR **(D)**, rDOR **(E)**, and mDOR **(F)**. A representative summation is shown (*n* ≥ 3).

We have previously established that systemic activation of the G_i/o_-coupled DOR with TAN-67, an agonist that only weakly recruits β-arrestin-2 to hDOR (Figure 3A), reduces voluntary intake in mice, but that SNC80, an hDOR agonist that strongly recruits β-arrestin-2 (Figure 3A) increases alcohol intake (28). However, we previously had not determined if a difference in receptor binding was observed between TAN-67 and SNC80 at hDOR to potentially explain differences in ligand bias. Using a SNAP-tag HTRF^®^ (Cis-Bio) approach we found that hDOR, TAN-67 exhibited a pKi = 7.7 ± 0.1 and SNC80 a pKi = 7.2 ± 0.2, with pKi = 5.8 ± 0.1 for leu-enkephalin (Figure 3B), suggesting that the only clear difference between TAN-67 and SNC80 is β-arrestin-2 recruitment efficacy. The surprisingly low affinity observed for leu-enkephalin may be an artifact of the fluorescent binding assay that relies on a large N-terminal SNAP-tag, which may potential interfere with the binding of relatively large peptide ligand, such as leu-enkephalin, but not small molecules.

Expanding from our previous study, we determined the equiactive bias factors for TAN-67 and SNC80 at hDOR using leu-enkephalin as a reference ligand (47) for β-arrestin-2 recruitment compared with G_i/o-_stimulated cAMP inhibition (a more positive bias factor = indicative of bias toward β-arrestin-2, more negative bias factor = indicative of bias toward cAMP activity). TAN-67 displayed a bias factor of −1.4 (cAMP biased) versus a +0.85 bias factor for SNC80 (β-arrestin-2-biased) (Figure 3C). To estimate what concentration of SNC80 to infuse *in vivo*, we relied on the Nielsen et al. reported findings in rat (25). Our *in vitro* assays suggest minimal differences in cAMP inhibition between human hDOR (Figure 3D), rat rDOR (Figure 3E), and mDOR (Figure 3F) for SNC80 (pIC_50_ = 7.8 ± 0.3, *n* = 3, pIC_50_ = 8.4 ± 0.1, *n* = 5, pIC_50_ = 8.4 ± 0.4, *n* = 3, respectively) and leu-enkephalin (pIC_50_ = 8.7 ± 0.2, *n* = 5, pIC_50_ = 8.9 ± 0.2, *n* = 5, pIC_50_ = 8.3 ± 0.1, *n* = 6, respectively). Because the affinity and efficacy of TAN-67 is very comparably to SNC80 (Figures 3A,B), we decided to infuse 10 nM TAN-67 and SNC80 into the mouse dorsal striatum to investigate the role of G_i/o_ signaling versus β-arrestin-2 recruitment in the modulation of alcohol use.

### Differential Modulation of Alcohol Intake Following Dorsal Striatal DOR Activation by G_i/o_-Biased Versus β-Arrestin-2-Biased DOR Agonists

**Figure 4:**
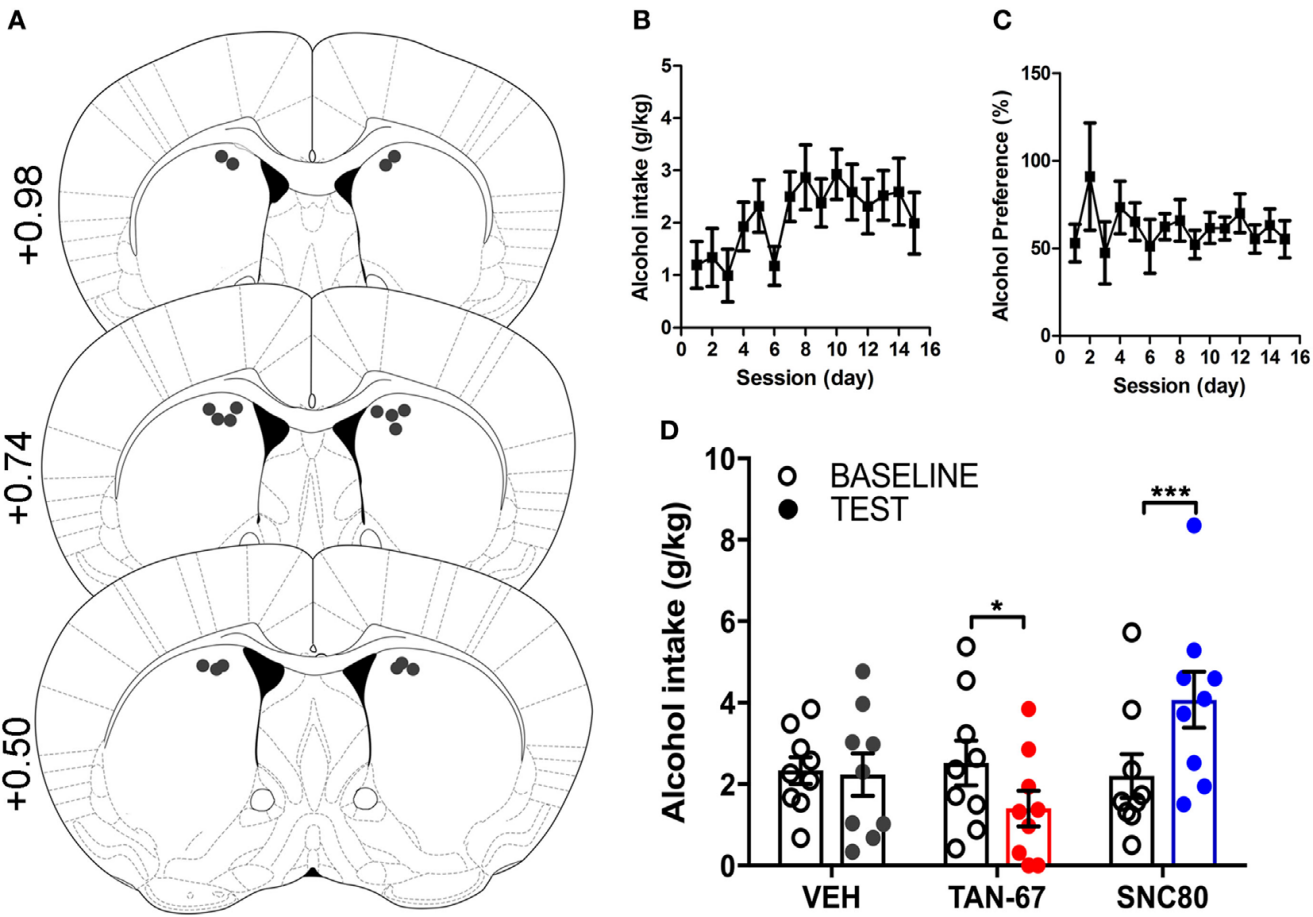
Dorsal striatal infusion of G_i/o_-protein biased delta-opioid receptor (DOR) agonist TAN-67 decreases voluntary alcohol intake, while β-arrestin-2 biased DOR agonist SNC80 increases alcohol intake in wild-type mice. Cannula placement was verified for all animals included in behavioral analysis **(A)**. C57BL/6 male, wild-type mice (*n* = 9–10) were trained to consume 10% alcohol over the course of 3 weeks, during which they increased their alcohol intake **(B)** and alcohol preference **(C)**. Vehicle saline (0.9%) infusion did not change alcohol intake while TAN-67 (10 µM) significantly decreased alcohol intake and SNC80 (10 µM) significantly increased alcohol intake **(D)**. Significance by repeated measures, multiple comparisons (Tukey) two-way ANOVA, *p < 0.05 and ***p < 0.001.

Cannula terminus location and patency were validated *via* trypan blue dye infusion into the dorsal striatum upon experimental completion (Figure 4A). Wild-type male animals (*n* = 9–10) were successfully trained to consume alcohol using a limited-access, two-bottle-choice (water versus 10% alcohol), DID protocol as shown by increased daily alcohol intake and preference (Figures 4B,C) compared with water intake (Figure S2A in Supplementary Material). For intra-striatal infusions, a significant drug (*p* = 0.03, see Table 2 for full statistical analysis for experimental group and Table S1 in Supplementary Material for Tukey comparisons between infusion weeks) and drug × test session effect (*p* < 0.0001) was observed, with no effect of test session alone, where Tukey multiple comparisons test revealed that 10 µM of TAN-67 significantly decreased voluntary alcohol intake (*p* = 0.04) while 10 µM SNC80 significantly increased alcohol intake (*p* = 0.0005). Importantly, vehicle (saline 0.9%) infusion did not affect alcohol intake (Figure 4D). No changes in water intake or alcohol preference were noted during these drug infusion testing sessions (Figures S2B,C in Supplementary Material).

**Table 2:** Two-way, repeated measures ANOVA of alcohol-related behaviors in wild-type mice upon biased delta-opioid receptor agonist infusion in the dorsal striatum.

Two-way ANOVA	df	Drug injection, alcohol intake	Drug injection, water intake	Drug injection, alcohol preference
Drug	2, 16	*F* = 4.38 *p* = 0.03	*F* = 0.63 *p* = 0.56	*F* = 1.40 *p* = 0.28
Test session	1, 8	*F* = 0.39 *p* = 0.55	*F* = 2.11 *p* = 0.18	*F* = 0.33 *p* = 0.58
Drug × test session	2, 16	*F* = 20.22 *p* < 0.0001	*F* = 0.49 *p* = 0.62	*F* = 1.39 *p* = 0.28
Multiple comparisons (Tukey)		VEH *p* > 0.99 TAN-67 *p* = 0.042 SNC80 *p* = 0.0005	VEH *p* > 0.96 TAN-67 *p* > 0.99 SNC80 *p* = 0.54	VEH *p* > 0.97 TAN-67 *p* = 0.85 SNC80 *p* = 0.88

### Genetic KO of β-Arrestin-2 Provides Additional Support for the Critical Role of DOR-Mediated G_i/o_-Coupling in the Dorsal Striatum in Decreasing Alcohol Intake

**Figure 5:**
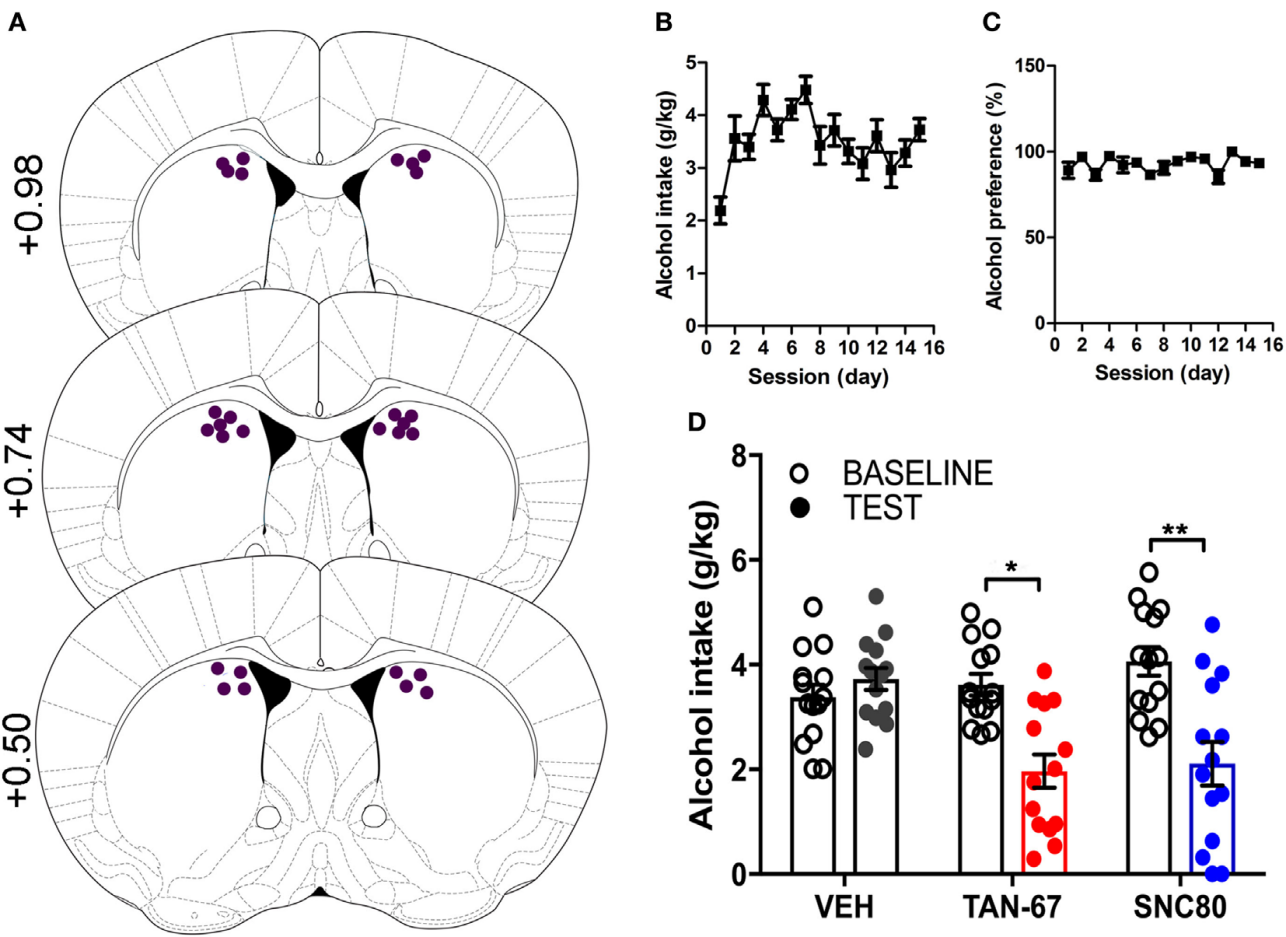
Genetic knockout (KO) of β-arrestin-2 reveals critical role of G_i/o_ signaling in reducing alcohol intake *via* dorsal striatal delta-opioid receptor activation. Cannula placement was verified for all animals included in behavioral analysis **(A)**. C57BL/6 male, β-arrestin-2 KO mice (*n* = 12) were trained to consume 10% alcohol over the course of 3 weeks, during which they increased their alcohol intake **(B)** and alcohol preference **(C)**. Vehicle saline (0.9%) infusion did not change alcohol intake, but both TAN-67 and SNC80 (10 µM) significantly decreased alcohol intake **(D)**. Significance by repeated measures, multiple comparisons (Tukey) by two-way ANOVA, *p < 0.05 and **p < 0.01.

β-Arrestin-2 KO male C57Bl/6 mice (*n* = 12) were surgically implanted with a bilateral cannula into the dorsal striatum before alcohol training, and cannula terminus location and patency were validated *via* trypan blue dye infusion upon experimental completion (Figure 5A). KO animals were successfully trained to consume alcohol using a limited-access, two-bottle-choice (water versus 10% alcohol), DID protocol (Figures 5B,C) compared with water intake (Figure S3A in Supplementary Material). A significant effect of drug (*p* = 0.003, see Table 3 for full statistical analysis for experimental group and Table S2 in Supplementary Material for Tukey comparisons between infusion weeks), test session (*p* = 0.002), and drug × test session (*p* = 0.0021) was identified for intra-dorsal striatal infusions, where multiple comparisons test found no effect of vehicle (saline 0.9%, *p* = 0.968) on alcohol intake. 10 µM of TAN-67 significantly decreased voluntary alcohol intake (*p* = 0.0113), and 10 µM of SNC80 also significantly decreased alcohol intake (*p* = 0.0021) (Figure 5D). This decrease was in contrast with that observed in wild-type animals and is the first report of SNC80’s ability to decrease voluntary alcohol intake, further suggesting that β-arrestin-2 functionality is key for SNC80-increased voluntary alcohol intake. No changes in water intake were noted during testing periods (Figure S3B in Supplementary Material), but a decrease in alcohol preference was noted for SNC80 infusion (*p* = 0.0018, Figure S3C in Supplementary Material). We have previously observed hyperlocomotion upon systemic SNC80 administration in both wild-type and β-arrestin-2 KO mice with increased alcohol intake or no change in alcohol intake, respectively (28, 48). Therefore, we questioned whether the decrease in alcohol intake upon dorsal striatal SNC80 infusion in the β-arrestin-2 KO was the result of changes in locomotion. However, SNC80 (10 µM) infusion into the dorsal striatum of β-arrestin-2 KO animals did not cause hyperlocomotion compared with vehicle infusion [Figures S4A,B in Supplementary Material, paired two-tailed Student’s *t*-test: *t*(6) = 1.68, *p* = 0.14], although the trend (albeit not significant) toward a decrease in locomotor activity suggests that there may be a potential influence of SNC80 on locomotor activity with respect to the decrease in alcohol intake observed upon SNC80 infusion in β-arrestin-2 KO animals.

**Table 3:** Two-way, repeated measures ANOVA of alcohol-related behaviors in β-arrestin-2 knockout mice upon biased delta-opioid receptor agonist infusion in the dorsal striatum.

Two-way ANOVA	df	Drug injection, alcohol intake	Drug injection, water intake	Drug injection, alcohol preference
Drug	2, 26	*F* = 7.31 *p* = 0.003	*F* = 1.07 *p* = 0.36	*F* = 3.11 *p* = 0.062
Test session	1, 13	*F* = 25.28 *p* = 0.0002	*F* = 0.93 *p* = 0.35	*F* = 7.19 *p* = 0.019
Drug × test session	2, 26	*F* = 7.92 *p* = 0.0021	*F* = 2.46 *p* = 0.11	*F* = 4.03 *p* = 0.03
Multiple comparisons (Tukey)		VEH *p* > 0.97 TAN-67 *p* = 0.011 SNC80 *p* = 0.0021	VEH *p* > 0.99 TAN-67 *p* = 0.95 SNC80 *p* = 0.28	VEH *p* > 0.99 TAN-67 *p* > 0.99 SNC80 *p* = 0.018

## Discussion

Through both chemogenetic and pharmacologic activation of G_i/o_-protein signaling, we observed that activation of G_i/o_-protein-coupled receptors in the dorsal striatum significantly decreases alcohol intake in male C57BL/6 mice by either inhibitory DREADD activation or activation of endogenously expressed DORs using a G-protein biased agonist. We specifically targeted the dorsal striatum as it plays an important role in modulating habitual alcohol use (2, 3, 7, 9), has strong DOR expression (20), and, crucially, is a region where DOR agonist SNC80 has been shown to increase alcohol intake in rats (25). Here, activation of virally expressed G_i/o_-coupled DREADDs in the dorsal striatum was capable of decreasing alcohol intake while no effect was observed in control-GFP animals upon CNO administration (Figure 1). For activation of endogenous dorsal striatal DORs, our findings that local dorsal striatal infusion of TAN-67 decreased alcohol intake and SNC80 increased alcohol intake (Figure 4; Figure S3 in Supplementary Material) agreed with our systemic findings (28) and also confirmed the previously observed alcohol intake increase following local dorsal striatal infusion of SNC80 in rats (25). Furthermore, through the use of β-arrestin-2 KO mice, we were able to shift the direction of alcohol intake modulation by SNC80 from significantly increasing intake to significantly decreasing consumption when β-arrestin-2 signaling pathways is not present (Figure 5; Figure S3 in Supplementary Material). This was expected as TAN-67 and SNC80 displayed similar binding and G-protein pathway efficacy at DOR *in vitro*, suggesting that the removal of potential β-arrestin-2 recruitment would allow the agonists to behave similarly (Figure 3). This shift is in agreement with our hypothesis that DOR-mediated G_i/o_ signaling is a potential strategy to reduce alcohol use, whereas DOR-mediated β-arrestin signaling is to be avoided.

While the dorsal striatum as a region in general is implicated in procedural learning (49–51), the dorsolateral striatum subregion is heavily associated with habitual behavior (behavioral actions that persist despite reward devaluation) (52) and the dorsomedial striatum with goal-directed learning (53). Chronic alcohol exposure may preferentially activate the dorsolateral striatum versus the dorsomedial striatum, as observed by increased glutamatergic transmission (54) and decreased GABAergic transmission (54, 55) in this subregion in animals exposed to chronic intermittent alcohol. Moreover, in rats, alcohol self-administration upregulates brain-derived neurotrophic factor (BDNF) in both the DLS and DMS, but with more robust increases in BDNF in the DLS (56, 57). Furthermore, infusion of BDNF in the DLS decreases alcohol self-administration (57). In rats, initial alcohol seeking was attenuated upon inactivation of the DMS (with no effect upon inactivation of the DLS). However, upon longer exposure to operant alcohol training, animals became insensitive to alcohol devaluation, and inactivation of the DLS re-sensitizes the animals to devaluation (6). Our results presented here did not differentiate between the DMS and the DLS, although future studies warrant investigation of G_i/o_-protein activity in these dorsal striatal subregions for potential subregion-specific differences in alcohol intake upon G_i/o_-protein activation.

To broadly validate the role of the dorsal striatum in alcohol consumption, we first virally expressed a G_i/o_-coupled DREADD (hM_4_Di) to artificially activate G_i/o_-protein signaling pathways in this region to determine how increased G_i/o_-protein activity altered alcohol intake. In the present study, activation by the hM_4_Di DREADD ligand CNO decreased alcohol intake of animals expressing hM_4_Di in the dorsal striatum and had no effect on control-GFP animals (Figure 1). Despite recent concerns on the use of DREADD technology and CNO’s conversion to clozapine *in vivo*, the low dose of 2 mg/kg was specific in its behavioral effects on the hM_4_Di-expressing mice compared with control GFP-expressing animals in drinking behavior (35), thus ruling out the potential issue that decreased consumption resulted from CNO (or clozapine following CNO conversion) activating endogenous muscarinic M_4_ receptors, which are also highly expressed in the striatum (58). In addition, as previously mentioned, no differences in locomotor activity were observed upon CNO administration in either control or DREADD-expressing mice, suggesting that the observed decrease in consumption did not result from hypolocomotion (Figure 2). Our viral AAV8-DREADD construct was expressed under a human synapsin promoter which specifically targets neurons (59), and given that the majority of the dorsal striatum consists of MSNs and AAV8 has been shown to infect GABAergic neurons in the mouse striatum (60), activation of virally expressed striatal hM_4_Di receptors in our experimental design likely inhibited both the D_1_R-MSNs and D_2_R-MSNs of the direct and indirect pathways, respectively. This net inhibition may be responsible for the observed no net change in locomotor activity and a modest—albeit significant—decrease in alcohol intake (3, 6, 8, 35, 41). We did not verify the potential of preferential tropism of the AAV8-DREADD construct [although AAV8 transduction in the striatum suggests that serotype 8 successfully transduces GABAergic neurons in the mouse striatum (60, 61)], thus limiting our conclusions on the specificity of increased G_i/o_-protein activity by DREADD activation on striatal GABAergic and/or cholinergic neurons. Furthermore, while our DREADD strategy was successful in confirming that inhibition of dorsal striatum by increased G_i/o_-protein signaling can decrease alcohol consumption, CNO is known to be an unbiased ligand for DREADD receptors (62, 63). Therefore, we next continued with an approach where we could more selectively activate endogenous G_i/o_-protein signaling over β-arrestin pathways.

Because of the limitations of potential tropism and possible β-arrestin-2 recruitment in our DREADD strategy, we next investigated changes in alcohol intake upon activation of G_i/o_-protein activity by infusing DOR agonists into the dorsal striatum, where DORs are endogenously expressed presynaptically on corticostriatal glutamatergic inputs (19), pre- and postsynaptically on cholinergic interneurons, and on D_2_-MSNs (20–22). In designing our DOR drug infusion experiments, we infused known DOR agonists into the dorsal striatum of either wild-type or β-arrestin-2 KO mice once a week (following 3 weeks of alcohol drinking) to assess changes in voluntary alcohol intake in response to drug infusion. In the first infusion test week, we infused vehicle (saline 0.9%) to ensure that handling and infusion alone did not change voluntary alcohol intake (Figures 4 and 5). In the second infusion test week, TAN-67 was infused, followed by SNC80 infusion in the third infusion test week. This specific order of drug infusion was determined based upon the *in vitro* β-arrestin-2 recruitment profiles of TAN-67 and SNC80 (Figure 3) and previously published work on SNC80’s ability to cause rapid DOR internalization [and potential degradation (64)] *in vitro* and *in vivo* (in the striatum) (20, 37). Thus, we infused TAN-67 first to prevent potential SNC80-induced desensitization of the DOR system and we did not counterbalance our drug infusions, thus limiting our conclusions on how observed SNC80 responses may be confounded by potential inflammation upon repeated drug infusion into this brain region. Because we specifically observe different behavioral effects with SNC80, which was injected last in both wild-type and β-arrestin-2 KO mice, we would argue that the observed responses represent a true pharmacological effect and are not a negative or positive consequence of repeated infusions.

Our findings that activation of G_i/o_ signaling in the dorsal striatum reduces alcohol intake would suggest a role for adenylyl cyclase and cAMP in this behavior. Recently, reductions in cAMP levels in the dorsal striatum by adenylyl cyclase type 1 (AC1) inhibition and AC1-KO have been associated with decreased ethanol-induced locomotor sensitization (65). Furthermore, blockade of dorsal striatal G_s_-coupled dopamine D_1_ receptors (but not blockade of G_i/o_-coupled dopamine D_2_) attenuates alcohol consumption (8), suggesting indeed that inhibition of cAMP production in the dorsal striatum may contribute to reduced alcohol use. In the dorsal striatum, alcohol can induce LTD of fast spiking interneuron-medium spiny neuron synapses *via* a mechanism involving DORs, as this LTD was blocked by a DOR antagonist and the effect was mimicked when using the DOR agonist DPDPE (24). Moreover, the effects of DPDPE can also be blocked by activating adenylyl cyclases with forskolin (24). In our hands, we find that DPDPE is relatively unbiased and thus also efficiently recruits β-arrestin (28). This may be relevant as it has been shown that LTD may also rely on functional β-arrestin-2 expression: activation of hippocampal metabotropic glutamatergic receptors attenuated LTD in β-arrestin-2 KO animals (66, 67) and, upon metabotropic glutamate receptor activation, β-arrestin-2 scaffolding proteins increase the synaptic strength of hippocampal neurons (68). Currently, no studies have investigated the role of β-arrestin-2 in alcohol and DOR-mediated LTD in the dorsal striatum, nor have studies investigated if contributions of cAMP and β-arrestin to LTD change in alcohol-exposed or alcohol-dependent animals.

The observation that β-arrestin-2 activation in the dorsal striatum increases alcohol intake in mice is in agreement with reported elevated expression levels of β-arrestin-2 gene (*Arrb2*) and β-arrestin-2 protein levels in the striatum of ethanol-preferring alko alcohol rats in comparison with alko non-alcohol rat counterparts, as well as decreased voluntary alcohol intake in β-arrestin-2 KO (69). Despite these connections of β-arrestin expression and voluntary alcohol intake, conflicting results exist on how alcohol intake is altered in β-arrestin-2 KO animals. Li et al. (70) observed that their β-arrestin-2 KO mice displayed increased voluntary alcohol consumption compared with wild-type mice, in line with behavior by our β-arrestin-2 KO mice which also showed slightly higher alcohol intake than wild-type mice (Figure 5) (28). One potential explanation is that the Björk et al. study used alcohol solutions that contained saccharin (28, 69, 70). Importantly, as a number of these aforementioned studies (including ours presented here) utilize global β-arrestin-2 KO animal models, we are limited in our interpretation on how global β-arrestin-2 expression affects general alcohol behavior because of potential compensatory expression of the β-arrestin-1 isoform, particularly because isoform-selective differences in behavior have been observed (71, 72). The effect of β-arrestin expression on alcohol intake is noteworthy as altered levels of β-arrestin-2 have been observed as a result of acute and/or chronic morphine exposure in rats (73), elevated glucocorticoid activity *in vitro* (74), during inflammation *in vivo* in synoviocytes, and after cerebral hypoxia/ischemia (75). It is possible that alcohol intake and preference by subjects in these situations is enhanced, and that effectiveness of therapeutic drugs may be altered in these subjects, i.e., an unbiased drug may become β-arrestin-biased and increase alcohol use.

The dorsal striatum contains a large variety of G_i/o_-coupled GPCRs besides DORs, including the muscarinic M_4_ and serotonin 5-HT_1B_ receptors (10, 11). In line with our current findings, all three G_i/o_-coupled receptors the respective KO animals (DOR KO, M_4_R KO, and 5-HT_1B_ KO mice) consume more alcohol compared with wild-type littermates (18, 76, 77). Here, our findings indicate that activation of dorsal striatal G_i/o_-coupled receptors, either *via* endogenous DORs or by virally expressed DREADDs, is sufficient to decrease voluntary alcohol intake in C57Bl/6 male mice. As β-arrestin-2 recruitment is associated with rapid internalization of DORs *in vitro* and *in vivo* [where DORs are degraded upon internalization (36, 37, 64)], we hypothesize that β-arrestin-2 recruitment to DORs by SNC80 can lead to rapid desensitization of endogenously expressed DORs, resulting in increased alcohol similar to that observed in DOR KO mice (18). In addition, SNC80-induced β-arrestin-2 recruitment may lead to β-arrestin-dependent signaling events (78), such as increased phosphorylation of ERK (79, 80). Previously, we discovered that agonists of the G_i/o_-coupled DOR can either decrease or increase alcohol intake in mice (29, 81, 82), and closer examination of the pharmacology of the DOR agonists revealed that agonists that strongly recruit β-arrestin-2 increased alcohol intake, whereas agonists that were G_i/o_-protein biased decreased alcohol intake in mice (28), suggesting that G_i/o_-protein biased ligands may be a therapeutic option in treating AUD. Combined with our current results, these studies suggest a potentially broad role for striatal G_i/o_-coupled signaling to decrease alcohol intake, which could be accomplished *via* G-protein biased ligands that activate G_i/o_-coupled receptors robustly expressed in the dorsal striatum, such as the DOR. Therefore, the development of G_i/o_-protein biased DOR agonists or agonists for other striatal G_i/o_-coupled receptors, such as the M_4_, 5-HT_1b_, dopamine D_2_ (83), kappa-opioid (84), and/or GPR88 receptor (85), could present a novel strategy to treat AUD by decreasing excessive alcohol consumption.

## Ethics Statement

This study was carried out in accordance with the recommendations of the National Institutes of Health Guide for the Care and Use of Laboratory Animals. The protocol (#1305000864) was approved by the Purdue University Institutional Animal Care and Use Committee.

## Author Contributions

MR performed cannulation surgeries, alcohol intake studies, locomotor studies, perfusions, cocaine challenge, and immunohistochemistry, and wrote the main draft of the manuscript. TC performed alcohol intake studies and bred and genotyped β-arrestin-2 KO mice. KM cloned the rDOR construct and performed the *in vitro* assays. DA performed locomotor studies and also bred and genotyped β-arrestin-2 KO mice. RR designed the experiments, assisted with the *in vitro* assays, cannulation surgeries, and alcohol intake studies, and wrote the manuscript. All the authors analyzed and interpreted data and proofread the final manuscript.

## Conflict of Interest Statement

The authors declare that the research was conducted in the absence of any commercial or financial relationships that could be construed as a potential conflict of interest.
