# Supplementary material for: Critical Role for Gi/o-Protein Activity in the Dorsal Striatum in the Reduction of Voluntary Alcohol Intake in C57Bl/6 Mice
Source: Front Psychiatry. 2018 Apr 5;9:112. doi: 10.3389/fpsyt.2018.00112 (PMC5900748; doi:10.3389/fpsyt.2018.00112)
Supplement: Supplementary file 1 [file image_1.PDF]

Supplementary Material

Critical role for Gi/o-protein activity in the dorsal striatum in the reduction of voluntary alcohol intake in C57Bl/6 mice

Meridith T. Robins[1], Terrance Chiang[1], Kendall L. Mores[1], Doungkamol Alongkronrusmee[1], Richard M. van Rijn[1*]

[1]Purdue University Institute for Integrative Neuroscience, Department of Medicinal Chemistry and Molecular Pharmacology, Purdue University, West Lafayette, Indiana 47907

*** Correspondence:**
Corresponding Author
rvanrijn@purdue.edu

1 Supplementary Figures

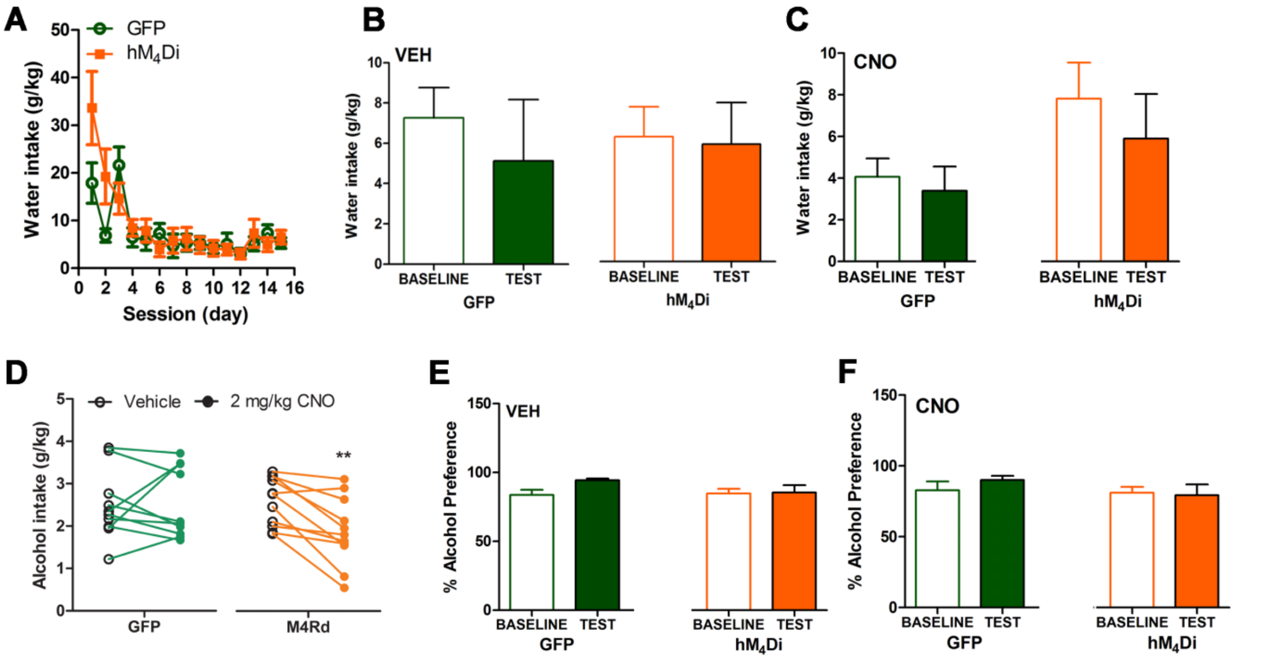

Supplemental Figure 1. CNO activation of striatal M_4RDi does not change water intake or alcohol preference.
Daily water intake (**A**) was recorded during the four-hour, DID sessions over the three training weeks (n=10-11 per group). No significant changes in water intake were observed after vehicle (**B**) or CNO (**C**) injection in either control-GFP or hM_4Di-mCherry expressing animals. Directional response in alcohol consumption for control-GFP or hM_4Di-mCherry expressing animals injected with CNO (**D**). Additionally, no changes in alcohol preference were observed after vehicle (**E**) or 2 mg/kg CNO injection (**F**) in either control-GFP or hM_4Di-mCherry expressing animals. Significance by unpaired, student's t-test for AUC or 2-way ANOVA with Bonferroni post-test for matching, **, $p<0.01$.

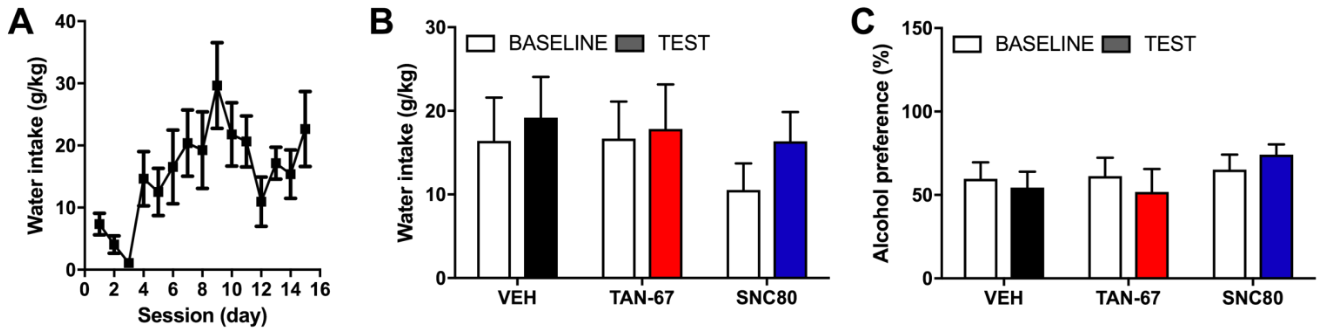

Supplemental Figure 2. Water intake during training and drug infusion sessions for wild-type mice receiving dorsal striatal drug infusions.
Daily water intake (**A**) was recorded during the four-hour, DID sessions over the three training weeks for wild-type, male C57Bl/6 animals (n=9-10). No significant changes in water intake (**B**) or alcohol preference (**C**) were observed during drug infusion sessions. Significance by repeated measures, multiple comparisons 2-way ANOVA.

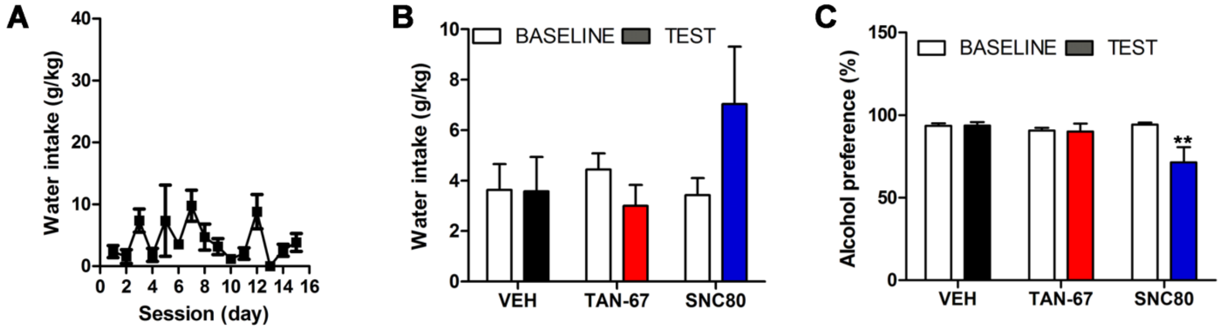

Supplemental Figure 3. Water intake during training and drug infusion sessions for β-arrestin-2 KO mice receiving dorsal striatal drug infusions.
Daily water intake (**A**) was recorded during the four-hour, DID sessions over the three training weeks for β-arrestin-2 KO animals (n=12). No significant changes in water intake (**B**) were observed during drug infusion sessions, yet a significant decrease in alcohol preference was found upon SNC80 infusion (**C**). Significance by repeated measures, multiple comparisons 2-way ANOVA, **, p<0.01.

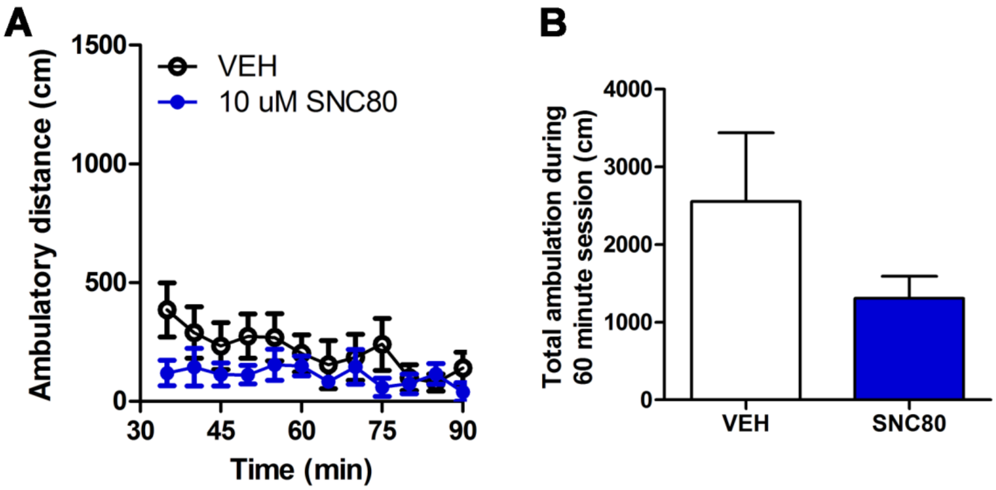

Supplemental Figure 4. Intra-dorsal striatal infusion of 10 µM SNC80 does not increase locomotor activity in β-arrestin-2 KO animals.

Locomotor activity of C57BL/6 male, β-arrestin-2 KO mice (n=7) was monitored 30 minutes after vehicle (0.9% saline) and SNC80 (10 µM) infusion for a total of 1 hour (**A**). No significant increase or decrease was observed upon SNC80 infusion (**B**). Statistics by paired two-tailed t-test.

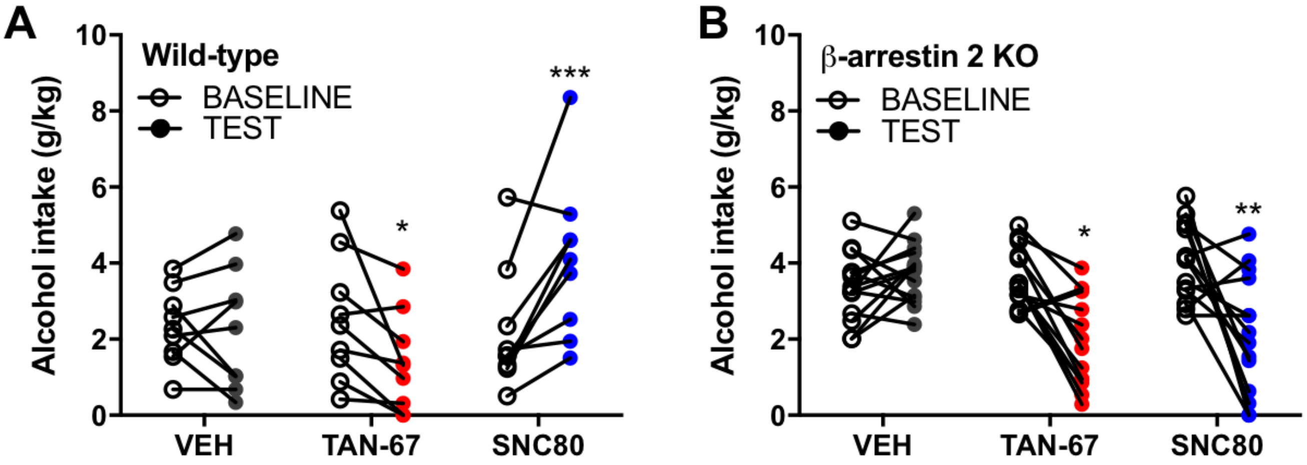

Supplemental Figure 5. Directional change in alcohol intake in both wild-type and β-arrestin-2 KO during following drug infusion.

In wild-type, C57Bl/6 male mice (n=9-10), vehicle saline (0.9%) infusion did not change alcohol intake while TAN-67 (10 µM) significantly decreased alcohol intake and SNC80 (10 µM) significantly increased alcohol intake (**A**). In β-arrestin-2 KO C57Bl/6 male mice (n=12), vehicle saline (0.9%) infusion did not change alcohol intake, but both TAN-67 and SNC80 (10 µM) significantly decreased alcohol intake (**B**). Significance by repeated measures, multiple comparisons (Tukey) by 2-way ANOVA, *, $p<0.05$, **, $p<0.01$, ***, $p<0.001$.

Supplemental Table 1. Tukey multiple comparisons test for alcohol intake between baseline and testing days for each infusion week for wild-type mice upon biased DOR agonist infusion in the dorsal striatum.

**BASELINE**	**Significance**	**Adjusted p-value**
VEH vs. TAN-67	**ns**	0.8526
VEH vs. SNC80	**ns**	0.9160
TAN-67 vs. SNC80	**ns**	0.6234
**TEST**	**Significance**	**Adjusted p-value**
VEH vs. TAN-67	**ns**	0.0619
VEH vs. SNC80	*******	0.0002
TAN-67 vs. SNC80	********	<0.0001

Supplemental Table 2. Tukey multiple comparisons test for alcohol intake between baseline and testing days for each infusion week for β-arrestin 2 knockout mice upon biased DOR agonist infusion in the dorsal striatum.

**BASELINE**	**Significance**	**Adjusted p-value**
VEH vs. TAN-67	**ns**	0.8517
VEH vs. SNC80	**ns**	0.2877
TAN-67 vs. SNC80	**ns**	0.5821
**TEST**	**Significance**	**Adjusted p-value**
VEH vs. TAN-67	**	0.0015
VEH vs. SNC80	**	0.0033
TAN-67 vs. SNC80	**ns**	0.9459
